# Acute appendicitis in a malpositioned appendix leading to retroperitoneal abscess and sepsis: a forensic autopsy case

**DOI:** 10.1007/s12024-025-01107-3

**Published:** 2025-10-23

**Authors:** Taketo Hashida, Sohtaro Mimasaka, Yuhei Matsuo, Maki Ohtani, Akira Hayakawa

**Affiliations:** 1 Akita University School of Medicine, Akita, Japan; 2 Department of Forensic Medicine, Tohoku University Graduate School of Medicine, 2-1 Seiryo-machi, Aoba-ku, Sendai, Miyagi 980-8575 Japan; 3 Department of Forensic Sciences, Akita University Graduate School of Medicine, Akita, Japan

**Keywords:** Malpositioning of the appendix, Acute appendicitis, Retroperitoneal abscess, Soft tissue infection, Sepsis

## Abstract

We report an autopsy case of a 74-year-old man with lower back pain who was referred to an orthopedic surgeon. Three days after he was prescribed acetaminophen for lumbar disc herniation, he was found dead in his bed. The autopsy revealed a perforated appendicitis attached to the retroperitoneum in the upper right abdomen, massive hemorrhages in the cecum, a retroperitoneal abscess, and skin discoloration from the abdomen to the back. The patient had died from sepsis caused by a retroperitoneal abscess and soft tissue infection secondary to perforated appendicitis adhering to the retroperitoneum. The C-reactive protein level was 43.5 mg/dL. Typically, perforated appendicitis causes peritonitis and sepsis. However, in our patient, intraperitoneal inflammation was limited to the region around the appendix, with no involvement of the surrounding intraperitoneal organs and no signs of peritonitis. Therefore, we speculated that appendiceal displacement was associated with retroperitoneal abscess. To our knowledge, this case study presents a rare case of retroperitoneal abscess caused by appendicitis.

## Introduction

Acute appendicitis is a common disease manifesting as acute abdominal pain [1]. Disease progression begins with catarrhal appendicitis, followed by phlegmonous appendicitis and gangrenous appendicitis, each increasing in severity [2]. Patients with catarrhal appendicitis present with visceral pain, vomiting, and anorexia [2]. Phlegmonous appendicitis manifests as somatic pain, muscular defense, fever, and elevated C-reactive protein (CRP) levels [2]. Patients with gangrenous appendicitis often present with peritonitis, leading to sepsis [2]. We report an autopsy case of a patient with a perforated and malpositioned appendix that did not cause peritonitis but resulted in a retroperitoneal abscess and sepsis. This is a forensically rare case. We also refer to 22 reported cases of retroperitoneal abscess with acute appendicitis.

## Case report

### Clinical history

A 74-year-old man was found dead in his bed. He was unemployed and lived alone. He had a history of hypertension, atrial fibrillation, lumbar disc herniation, and colonic polyps. Approximately 1 month before his death, he consulted his primary care physician for hypertension and atrial fibrillation, who prescribed amlodipine besilate and rivaroxaban. Ten days before his death, he refrained from alcohol and tobacco because he felt sick. He also mentioned experiencing abdominal pain and difficulty eating, which he attributed to his lower back pain. Three days before his death, he consulted a nearby orthopedic unit with a 2-week history of lower back pain. Radiographic and magnetic resonance imaging (MRI) findings confirmed the diagnosis of lumbar disc herniation, and he was prescribed acetaminophen for the pain. His friend and uncle visited him on the eve of his death and reported that his abdomen had appeared swollen. The following day, he was found in a prone position with partial lateral rotation by his sister-in-law. An ambulance crew confirmed his death upon arrival, noting rigor mortis, and lividity. He was not transported to the hospital.

### Autopsy findings

#### Postmortem imaging

**Fig. 1:**
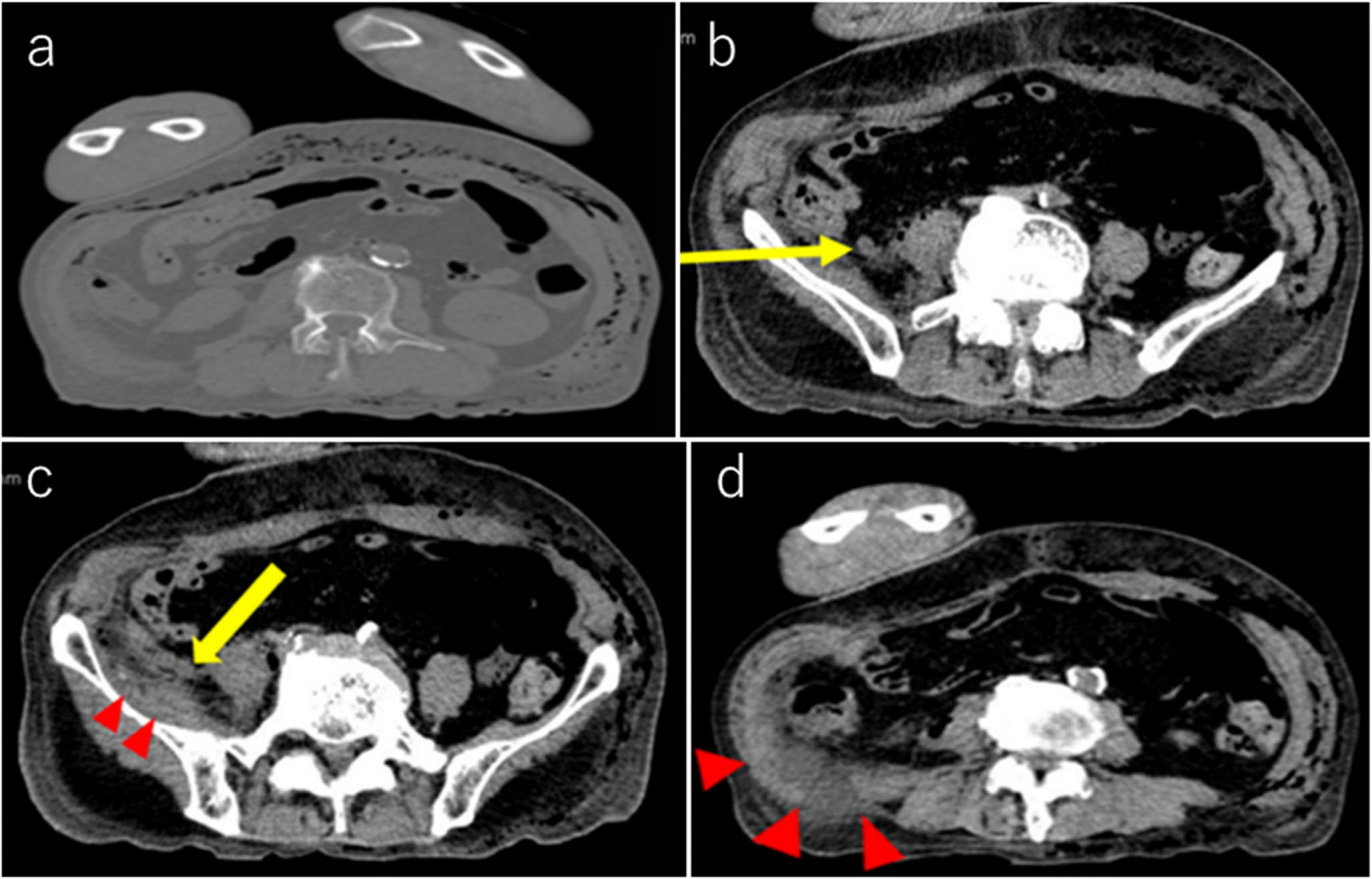
Axial view on postmortem computed tomography. **a** Free air was present in the subcutaneous abdominal region. **b** The appendix (yellow arrow) was observed in the right upper abdominal region. **c** A low-density area (red arrowhead) was found in the retroperitoneum. The appendix (yellow arrow) was observed next to the iliopsoas muscle. **d** A low-density area (red arrowhead) was found in the retroperitoneum

Before starting the autopsy, we performed plain computed tomography (CT). Free air was observed in the subcutaneous abdominal region in the axial view (Fig. 1a). The appendix (yellow arrow) was present in the right upper abdominal region in axial view (Fig. 1b) and was located adjacent to the iliopsoas muscle (Fig. 1c). A low-density area, indicative of an abscess (red arrowhead), was observed in the retroperitoneum (Fig. 1d).

#### External examination

**Fig. 2:**
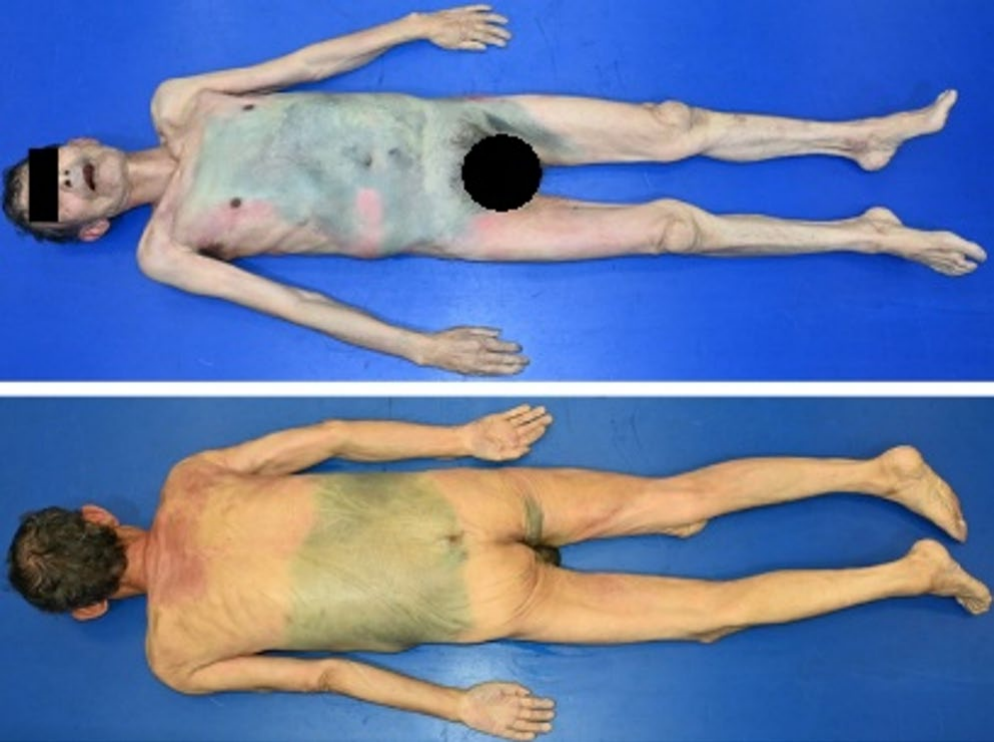
Findings of external examination. Greenish-brown skin discoloration was observed on the anterior and posterior surfaces and thighs

The deceased was a normally nourished man (weight: 55.0 kg; height: 164 cm). His skin was pale, with a moderate degree of reddish-purple lividity on the front and back (Fig. 2). Greenish-brown skin discoloration was seen on the anterior and posterior surfaces and thighs (Fig. 2). No traumatic injuries were apparent on external examination.

#### Internal examination

**Fig. 3:**
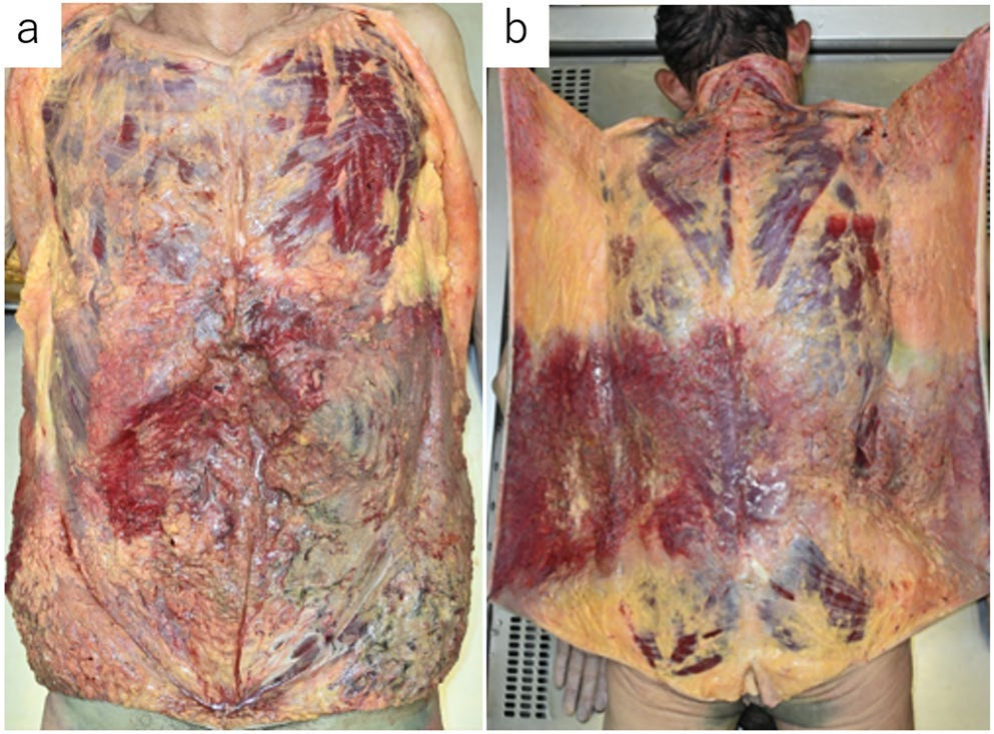
Internal examination. **a** In the anterior subcutaneous layer: Greenish-brown skin discoloration was observed at the site of soft tissue infection. **b** In the posterior subcutaneous region: Many subcutaneous hemorrhages and edematous areas were observed on the back

**Fig. 4:**
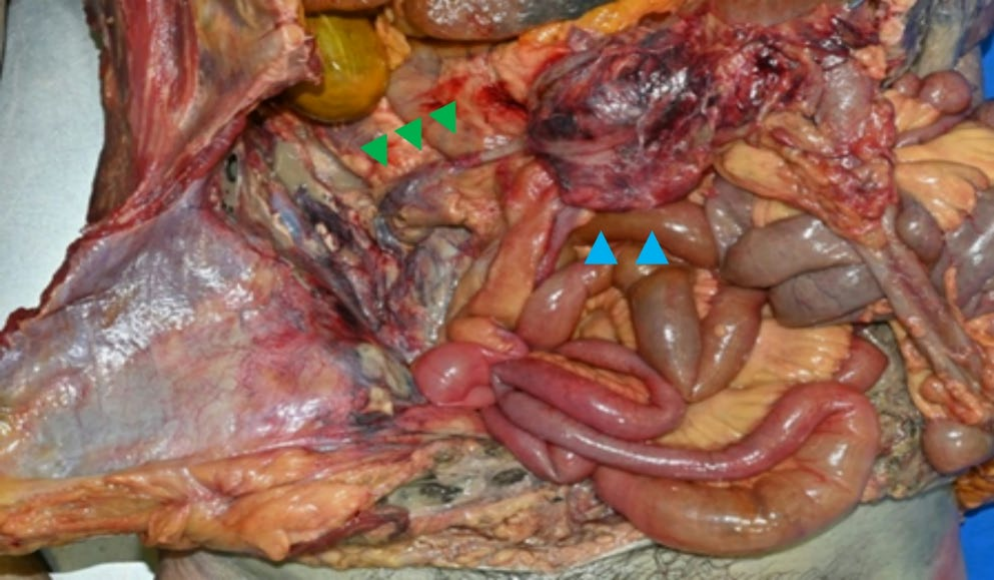
Internal examination of the abdomen. The cecum and appendix were observed in the right upper quadrant. Many hemorrhages were present on the cecum (light blue arrowhead). The tip of the appendix adhered to the retroperitoneum (green arrowhead)

**Fig. 5:**
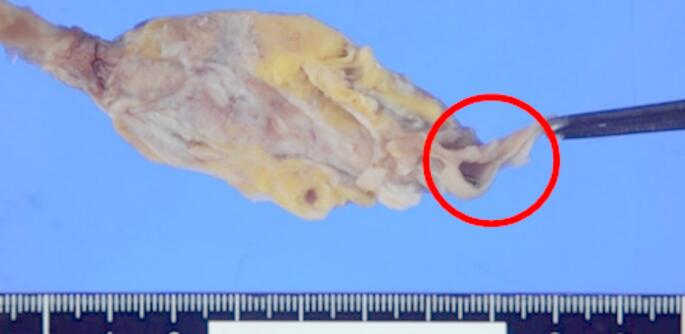
Internal examination of the appendix after formalin fixation. The tip of the appendix (red circle) was perforated

**Fig. 6:**
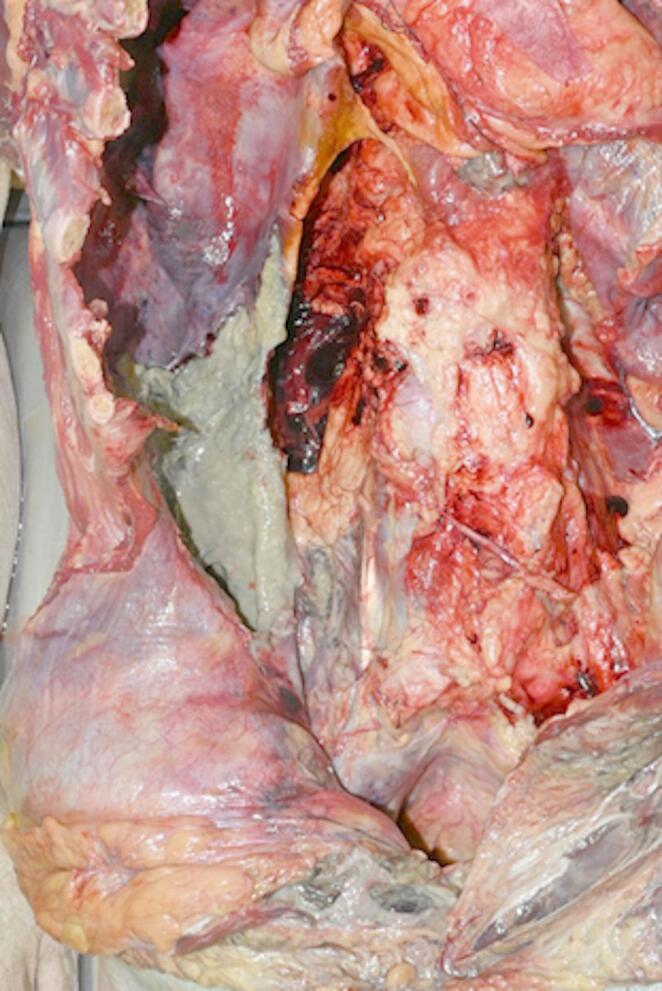
Internal examination of the right retroperitoneum. A massive abscess was observed in the retroperitoneum

Greenish-brown skin discoloration was observed in the area of soft tissue infection in the anterior subcutaneous region (Fig. 3a). No pericardial fluid, pleural effusion, or ascites was observed. The cecum and appendix were present in the upper right abdominal region (Fig. 4). Multiple hemorrhages were present in the cecum, and the tip of the appendix was adherent to the retroperitoneum. The perforated tip of the appendix (red circle) is shown in Fig. 5. Notably, a massive abscess was present in the retroperitoneum (Fig. 6). Additionally, numerous subcutaneous hemorrhages and edematous areas were observed on the back (Fig. 3b). Finally, chicken fat clots were observed in the cardiac blood.

#### Histopathological findings

**Fig. 7:**
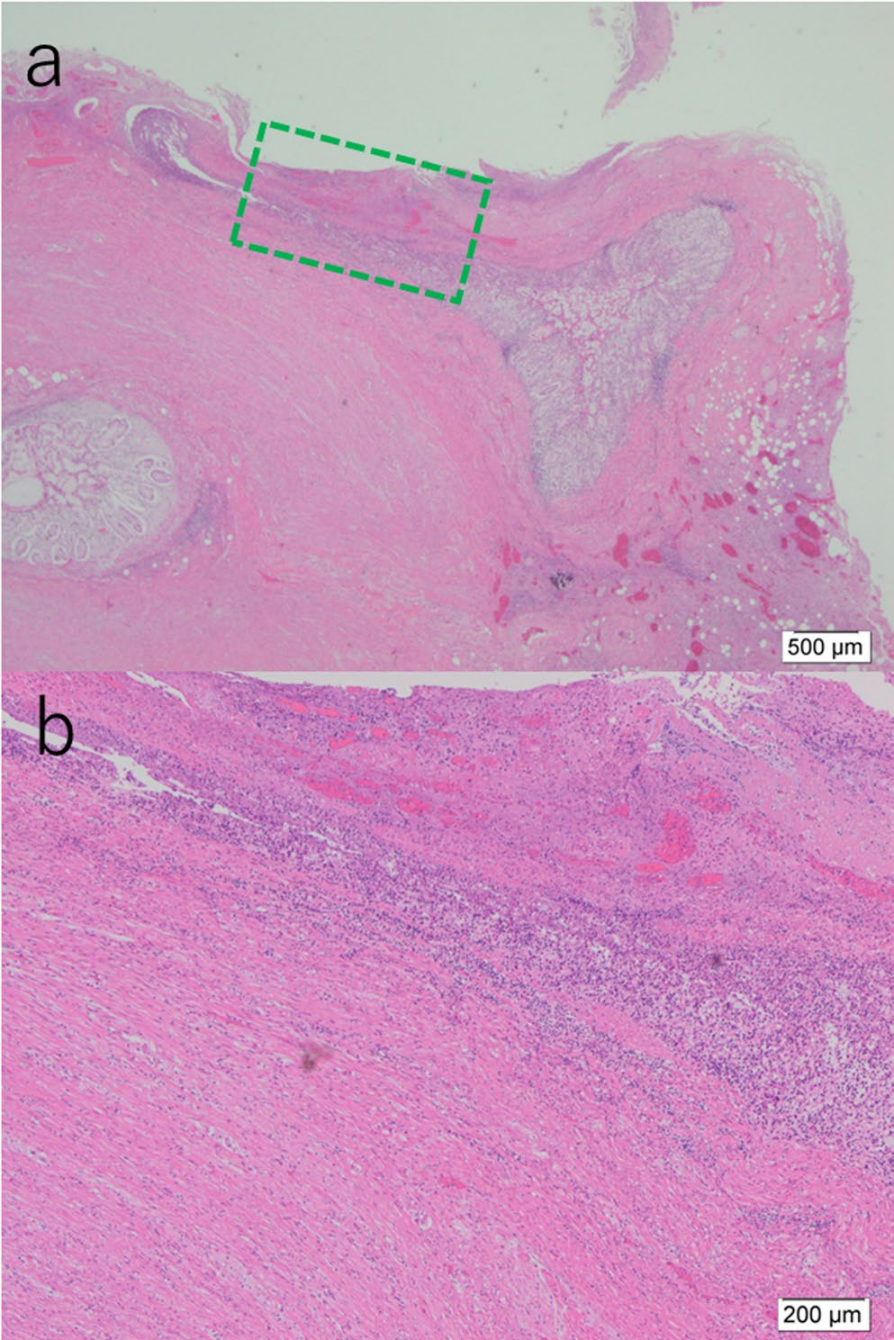
Histological examination showing inflammatory cells of the appendix (green dotted square) (hematoxylin and eosin; a) ×1.25, b) ×4)

Histological examination revealed the presence of inflammatory cells in the appendix (Fig. 7, green dotted square). The perforated part of the appendix was necrotic, and histological examination could not be performed. Microscopic examination revealed no abnormalities in the other organs.

#### Toxicology/microbiology studies

Ethyl alcohol was not detected in the heart blood, peripheral blood, or urine. The glycated hemoglobin concentration of cardiac blood was 5.8%. No drugs were detected on urine drug screening, conducted with the Triage DOA^®^ kit. However, a peripheral blood sample showed traces of amlodipine besilate and acetaminophen when tested for drugs using liquid chromatography-mass spectrometry. A pus sample from the right retroperitoneum revealed the following species on microbiological testing using culture and matrix-assisted laser desorption ionization–time of flight mass spectrometry: *Escherichia coli*, *Streptococcus* species, *Klebsiella pneumoniae*, *Bacteroides fragilis*, *Bacteroides nordii*, and *Fusobacterium* species.

#### Laboratory results

The CRP concentration in cardiac blood was 43.5 mg/dL.

## Discussion

This case demonstrates an unusual presentation of acute appendicitis, where malpositioned anatomy led to retroperitoneal abscess formation and fatal sepsis. The appendix is located near the root of the taenia coli at the surface of the cecum and is suspended by the mesoappendix, allowing for some mobility. Perforated appendix may lead to a retroperitoneal abscess, as contamination of the retroperitoneum through digestive tract perforation is a known cause of such abscesses [3]. Thus, our patient’s lower back pain could have been a symptom of retroperitoneal abscess [3]. The greenish-brown skin discoloration and presence of free air in the subcutaneous abdominal region were evidences of soft tissue infection. Furthermore, the cause of death could have been sepsis due to a retroperitoneal abscess. Microbiological testing can detect the causative bacteria of retroperitoneal abscess [3–5].

Several studies have reported on peritonitis due to acute appendicitis. However, it was unlikely that our patient had peritonitis given the absence of any obvious signs of inflammation and bleeding at the jejunum, ileum, and colon, except for the cecum and peritoneal area around the appendix (Fig. 4), as well as the absence of ascites in the abdominal cavity, which is a representative symptom of peritonitis [5].

Ileocecal abscess with acute appendicitis has been described in the literature [2]. We performed a literature search on retroperitoneal abscess with acute appendicitis using the Igaku Chuo Zasshi and PubMed databases. Shimizu et al. reported 17 cases (two patients died) in Japan between 1987 and 2006 [6], and Hsieh et al. reported 24 cases (four patients died due to severe sepsis) abroad between 1955 and 2005 [7]. Among studies published after 2006, we identified 22 reported cases of retroperitoneal abscess with acute appendicitis. A summary of these 22 cases and the current cases is provided in Table 1 [8–29]. In two of these cases, acute appendicitis caused soft tissue infection, extending from the right abdomen to the back, resulting in the formation of a retroperitoneal abscess and culminating in sepsis. One of the two patients succumbed to sepsis and multiple organ failure. However, both patients had acute appendicitis (in the lower right abdomen) without malpositioning of the appendix.

**Table 1:** Clinical findings in 23 cases of extensive abscess formation caused by perforated acute appendicitis

No.	Publication year	Age	Sex	Presenting symptoms	Location of appendicitis	Location of abscess	Outcome	References No.
1	2008	76	M	AP, Loss of Appetite	Retrop.	Retrop, abdominal Wall, Left Perirenal	Died	[8]
2	2009	77	M	AP, LBP	Retrocecal	Retroperitoneal Space	Cured	[9]
3	2009	34	M	RFP, Nausea, Vomiting, Loss of Appetite	Retrocecal	Retrocecal	Cured	[10]
4	2010	21	M	Back Pain	Retrop.	Retrop.	Cured	[11]
5	2010	43	M	AP, Nausea, Constipation	Retrop.	Retroperitoneal Space	Cured	[12]
6	2010	3	M	AP, Fever, Vomiting, Diarrhea	Ascending Colon	Back of the Ascending Colon	Cured	[13]
7	2010	60	M	RLP, Fever	Retrop.	Right perinephric Space	Unknown	[14]
8	2012	41	M	RLP, Right Thigh Pain	Retrocecal	Retrorenal and Retrocecal Retrop.	Cured	[15]
9	2012	78	F	Swelling and Pain in the Right Gluteal Region	Retrocecal	Retroperitoneal Iliopsoas Area	Unknown	[16]
10	2013	85	F	RFP, Right Thigh Pain, Fever	Retrocecal, Right Thigh	Right Retrop.~Right Thigh	Cured	[17]
11	2013	52	M	RLQ Pain, Fever, Diarrhea	Retrocecal	RKIP~RLQ	Cured	[18]
12	2013	53	M	Lower Abdominal Pain	Retrop.	Duodenum~Ascending Colon	Cured	[19]
13	2014	59	M	RLQ pain, Fever	Retrop.	Retrop.	Cured	[20]
14	2014	56	M	Right Hip Pain	Right Psoas Muscle	Psoas	Cured	[21]
15	2014	10	M	Fever, Diarrhea	Retrocecal	Right Anterior Pararenal Space	Cured	[22]
16	2015	22	F	AP, Fever, Nausea	Retrocecal	Retrop.	Cured	[23]
17	2015	50	M	RFP, Fever	Retrop.	Retrop.	Died	[24]
18	2019	50	F	RFP, Nausea, Vomiting	Retrocecal, subhepatic	Retrop.	Cured	[25]
19	2022	45	M	AP	Ileocecal Region	Pre-peritoneal~Retroperitoneal Space	Cured	[26]
20	2022	15	M	AP, CP, Cough, Fever	Retrocecal	Retrop, Right Lung	Cured	[27]
21	2023	70	M	RLQ pain, Fever	Unknown	Dorsal Ileocecal Region	Cured	[28]
22	2023	69	M	AP, Nausea, Vomiting	Retrocecal	Retroperitoneal Space	Cured	[29]
23	―	74	M	LBP	Right Upper Retrop.	Retroperitoneal Space	Died	―

The cause of malpositioning of the appendix in the upper right quadrant in our patient may have been previous abdominal surgery or congenital. We performed a literature search on congenital malpositioning of the appendix using the Igaku Chuo Zasshi and PubMed databases. Imaizumi et al. reported that one of the causes of acute appendicitis, albeit in the lower right abdomen, was intestinal malrotation, and 20 cases of the same were reported between 1983 and 2011 in Japan [30]. Dimitriadis et al. reported a case in which the appendix was located near the liver edge and acute appendicitis caused upper right abdominal pain [31]. We cannot completely reject the possibility that the patient had undergone abdominal surgery, although no surgical scars were evident during the autopsy. We speculated that the malpositioning of the appendix caused acute appendicitis in the upper right abdomen. Notably, no cause other than appendicitis was identified for the retroperitoneal abscess.

Our patient succumbed to sepsis. He probably had a congenitally malpositioned appendix and cecum caused by intestinal malrotation. He developed acute appendicitis in the right upper quadrant, resulting in an extensive retroperitoneal abscess, extending from the upper right abdomen to the lower right abdomen. The retroperitoneal abscess, in turn, caused extensive soft tissue infection, extending from the abdomen to the back. Similar cases have rarely been reported in the literature.

Given the position of the retroperitoneal abscess, the orthopedic physician did not consider diagnosing the patient with sepsis due to a retroperitoneal abscess caused by acute appendicitis in the upper right abdomen; the patient was misdiagnosed with lumbar disc herniation based on radiographic and MRI findings and prescribed acetaminophen. However, we could not find evidence of lumbar disc herniation on postmortem CT. Accurate diagnosis and consequent treatment of acute appendicitis and retroperitoneal abscess was further obscured because acetaminophen mitigated the fever and pain. Hypothetically, measurement of CRP at this stage would have revealed considerable elevation, and the misdiagnosis of lumbar disc herniation could have been averted.

Acute appendicitis in the upper right abdomen causes atypical symptoms, increasing the risk of misdiagnosis. Therefore, clinicians should consider acute appendicitis in the differential diagnosis of lower back pain and conduct thorough medical interviews.

### Limitations

This report describes a single case, with incomplete surgical history and limited ante-mortem clinical information. Future studies should investigate the prevalence and outcomes of retroperitoneal abscesses due to malpositioned appendices.

### Recommendations

Emergency and general physicians should suspect malpositioned appendicitis in patients with posterior or atypical abdominal pain. Comprehensive autopsy is essential in cases of unclear sepsis.

## Conclusion

We report an unusual case of a patient with intestinal malrotation who developed a retroperitoneal abscess due to acute appendicitis, resulting in soft tissue infection, sepsis, and eventual death. Older patients with acute appendicitis have no obvious symptoms, and the diagnosis is easily delayed, whereas patients with typical acute appendicitis usually present with rebound tenderness and guarding related to peritonitis. However, the peritoneal irritation sign is not always seen in cases of atypical appendicitis with retroperitoneal abscess. Such cases are rarely encountered in general practice, and the literature contains very few case reports of appendicitis with retroperitoneal abscess. Furthermore, diagnosing acute appendicitis in this case was particularly challenging because the cecum and appendix were located in the upper right abdomen. The cause of death was determined through the autopsy; therefore, the importance of autopsy should be realized. We propose that general medical textbooks should inform students and physicians that acute appendicitis can lead to the development of a retroperitoneal abscess.

## Conclusion

We report an unusual case of a patient with intestinal malrotation who developed a retroperitoneal abscess due to acute appendicitis, resulting in soft tissue infection, sepsis, and eventual death. Older patients with acute appendicitis have no obvious symptoms, and the diagnosis is easily delayed, whereas patients with typical acute appendicitis usually present with rebound tenderness and guarding related to peritonitis. However, the peritoneal irritation sign is not always seen in cases of atypical appendicitis with retroperitoneal abscess. Such cases are rarely encountered in general practice, and the literature contains very few case reports of appendicitis with retroperitoneal abscess. Furthermore, diagnosing acute appendicitis in this case was particularly challenging because the cecum and appendix were located in the upper right abdomen. The cause of death was determined through the autopsy; therefore, the importance of autopsy should be realized. We propose that general medical textbooks should inform students and physicians that acute appendicitis can lead to the development of a retroperitoneal abscess.

## Key points

1. The patient was found dead at his house 3 days after having consulted an orthopedic surgeon for lower back pain.
2. In the internal examination, perforated appendix attached to the retroperitoneum in the upper right abdomen, massive hemorrhages in the cecum, and a retroperitoneal abscess were observed.
3. Perforated appendicitis causes peritonitis and sepsis, but this patient died from sepsis caused by a retroperitoneal abscess and soft tissue infection secondary to perforated appendicitis adhering to the retroperitoneum.
4. We suspected that appendiceal displacement was associated with the retroperitoneal abscess; malpositioned appendix can result in retroperitoneal abscess and fatal sepsis due to delayed diagnosis.
5. Forensic autopsy is crucial for identifying atypical presentations in unclear sepsis cases. Physicians should suspect acute appendicitis in patients with posterior abdominal symptoms or nonspecific complaints.

## Data Availability

Due to the nature of this case report, supporting data is not available.
